# Anorexia nervosa and the COVID-19 pandemic among young people: a scoping review

**DOI:** 10.1186/s40337-023-00843-7

**Published:** 2023-07-20

**Authors:** Anna C. Schlissel, Tracy K. Richmond, Misha Eliasziw, Kristin Leonberg, Margie R. Skeer

**Affiliations:** 1 Department of Public Health and Community Medicine, Tufts University School of Medicine, 136 Harrison Avenue, Boston, MA 02111 USA; 2 Division of Adolescent Medicine, Boston Children’s Hospital, 6th Floor, 333 Longwood Avenue, Boston, MA 02115 USA; 3 Harvard Medical School, Pediatrics, 25 Shattuck Street, Boston, MA 02115 USA; 4 Gerald J. and Dorothy R. Friedman School of Nutrition Science and Policy, Jaharis Family Center for Biomedical and Nutrition Sciences, Tufts University, 150 Harrison Avenue, Boston, MA 02111 USA

**Keywords:** Anorexia nervosa, COVID-19, Adolescents, Young adults, Restrictive eating disorders

## Abstract

**Background:**

The extent to which the recent global COVID-19 Pandemic has impacted young people with restrictive eating disorders [i.e., anorexia nervosa (AN) and atypical anorexia nervosa (AAN)] is unclear. We conducted a scoping review of the literature to identify how the pandemic has impacted this population and to identify gaps in the current literature to inform future research efforts.

**Main body:**

We searched PubMed, EMBASE, the Web of Science, The Cochrane Library, PsycInfo, ProQuest Dissertations and Theses Global, LitCovid, Google Scholar, and relevant agency websites from 2019 to 2022. We included studies that focused on young people with AN/AAN globally. Of the 916 unduplicated articles screened, 17 articles met the inclusion criteria, reporting on 17 unique studies including 4,379 individuals. Three key findings were identified. First, an increase in hospitalizations related to eating disorders was found during COVID-19 among young people with AN and AAN. Multiple studies cited increased medical instability, even though the overall duration of disease was shorter compared to pre-pandemic levels. Second, changes in eating disorder-related symptomology during the pandemic were reported in this population, as well as poorer overall behavioral and mental health. Suggested reasons behind changes included boredom or minimal distraction from pathological thoughts, increased social isolation, increased social media and online use (e.g., reading blogs or watching YouTube), gym and school closures, changes in routines due to lockdowns and quarantines, and worries over gaining the “Quarantine 15”. Third, there was an increase in the use of telemedicine as a treatment modality for the treatment of AN. Challenges were reported by both clinicians and patients regardless of past experience using telemedicine. When compared to no treatment, telemedicine was recognized as the best option during COVID-19 lockdowns; however some individuals expressed the preference for in-person treatment and planned to return to it once it became available.

**Conclusion:**

The pandemic significantly impacted young people with restrictive eating disorders as seen by increased hospitalizations and requests for outpatient care. A primary driver of the changes in eating disorder symptomatology may be lockdowns and quarantines. Further research investigating how the series of lockdowns and re-openings impacted individuals with AN/AAN is warranted.

## Abstract (plain language summary)

Data collected from a scoping review of published peer-reviewed literature during the first two years of the COVID-19 pandemic have highlighted the impact that the global pandemic has had on young people with anorexia nervosa and atypical anorexia nervosa. We found an increase in medical hospitalizations related to eating disorders, changes in eating disorder-related symptomology as well as overall poorer behavioral and mental health among this population as a result of COVID-19. We also found an increase in the use of telemedicine as treatment modality, particularly during lockdowns, which sheds light on more diverse modalities for treatment. Further research investigating how the series of lockdowns and re-openings impacted individuals with AN/AAN is warranted.

## Background

The SARS-COVID-19 Global Pandemic (COVID-19) has impacted young people since its emergence in November 2019. Past epidemics, pandemics, and disasters have all been found to be associated with increased rates of post-traumatic stress disorder, depression, and anxiety among adolescents [1–7]. As such, it is not surprising that globally, adolescents have experienced higher rates of anxiety, depression, and stress due to the COVID-19 pandemic [1, 6]. Overall, poorer mental health in general has been reported among adolescents and young adults since the start of the pandemic [5, 8–15]. Furthermore, increased rates of eating disorders [16–18], including anorexia nervosa (AN) and atypical anorexia nervosa (AAN) [7, 19], and increased rates of more severe eating disorders symptomatology [20–24], have been thought to be influenced by limited socialization, increased isolation, cancelled sports and extra-curricular activities, increased time spent with family, interrupted or decreased access to health care, reduced or eliminated established schedules, and increased general stress caused by the COVID-19 pandemic, such as fears about catching the virus or having a family member die from the virus, concerns about family financial stability, to concerns about access to food [1, 9, 25, 26], which were all caused or exacerbated by the pandemic.

The Diagnostic and Statistical Manual of Mental Disorders, fifth edition (DSM-5) defines AN as a restrictive eating disorder where a caloric deficit leads to a low body weight combined with an intense fear of gaining weight or becoming fat to such a degree that it interferes with weight gain as well as a cognitive misalignment that may include a lack of recognition of seriousness of low bodyweight [27]. AAN includes the same criteria for AN, however, despite significant weight loss, weight remains in the healthy weight range or above. Furthermore, individuals with AAN experience the same characteristics and psychological symptoms, as well as comorbid mental health conditions, as individuals with AN [28],

The increased incidence and severity of presentation in individuals with AN/AAN is significant because of the health and psychological consequences of this illness. AN, a restrictive eating disorder, while rare with a 0.6% lifetime prevalence rate in the United States [29, 30], is accompanied by significant co-morbidities and a high mortality rate [31–35]. The highest incidences of AN occurs between ages 13–18 with 104 per 100,000 person-years, the average length of disease is 3.4 years, and approximately one-third of AN cases will relapse [29, 30, 36–38]. AAN is also rare with a 2.8% lifetime prevalence by age 20 [38]. The incidence rate of AAN is 366 per 100,000 person-years and the peak age occurs around 19–20 years and individuals often experience a longer duration of illness before diagnosis and may experience worse eating-disorder specific symptomatology [38–40]. Furthermore, among all mental health conditions, AN has the second highest mortality risk [41], second only to opioid use disorder. Approximately one in five individuals with AN die by suicide and the likelihood of early premature death is six times greater for individuals with AN compared to individuals without AN [41, 42]. Morbidity rates have been found to be similar between individuals with AN and AAN [33, 43]. Suicidal behaviors have also been found to be similar between individuals with AN and individuals with AAN even though individuals with AN can present with greater ED psychopathology [33, 43–45]. Additionally, having AN is associated with problems in people’s social lives, home relationships, and physical morbidity as well as poorer health-related quality of life compared to individuals without AN [46–49]. AN may also have long-term implications for the functioning of nearly every system in the body, and onset or relapse can quickly result in life-threatening situations [31–35].

The data gathered during the first two years of the COVID-19 pandemic have illuminated the impact of the pandemic on young people with AN/AAN. Though prior scoping and systematic reviews have addressed the effects of COVID-19 on adolescent mental health [6], or the pandemic’s impact on lifetime risk of eating disorders [7, 19], insufficient attention has been given to the effects of COVID-19 on the incidence and severity of illness of AN/AAN. Furthermore, no review has focused on how the COVID-19 pandemic has specifically impacted the pivotal period of adolescence and young adulthood among those with AN/AAN. Further information is needed as the pandemic will continue to have implications for eating disorder prevention efforts, work force strategies, and treatment in the near future. Therefore, the aim of the current study is to examine the impact of the COVID-19 pandemic on young people with AN/AAN by mapping the extant literature.

## Method

We conducted a novel scoping review to map the current understanding of the impact of the COVID-19 pandemic on young people with restrictive eating disorders, specifically AN/AAN. Scoping reviews are similar to systematic reviews but address exploratory research questions especially on new and emerging topics, which include identifying types of available evidence, mapping current evidence in a field, and identifying and analyzing knowledge gaps in research [50]. We chose to conduct a scoping review as opposed to a systematic review, as the impact that COVID-19 has had on young people with AN/AAN is unclear and we sought to map the available evidence and identify knowledge gaps.

We followed the methodology for conducting scoping reviews presented by Peters et al. [51] which built upon the original Methods published in 2005 by Arksey and O’Malley [52], as well as the updated guidance by Peters et al. [53]. Tricco et al.’s PRISMA Extension for Scoping Reviews checklist was used for reporting [36].

### Study selection criteria

Studies eligible for inclusion included original research studies, systematic reviews, and other grey literature, such as reports, working papers, or government documents, published between November 2019 and April 2022. We specified November 2019 as the starting month since that was when the SARS-CoV-2 virus first emerged in Wuhan, China. We searched for studies in peer reviewed journals and the gray literature without any restrictions on study design or country. Exclusion criteria were: (1) animal studies, (2) studies before November 2019, (3) young people (mean age under 30) were not a major focus of the article, (4) focus not specifically on restrictive eating disorders (AN/AAN specifically), and (5) the COVID-19 Pandemic was not addressed as a primary topic. Once full texts were obtained, we applied four additional exclusion criteria: (1) case studies of less than two individuals, (2) opinion or discussion pieces, (3) articles not in English, and (4) articles that only described study protocols.

### Data sources and searches

We identified published literature from PubMed, EMBASE, the Web of Science, The Cochrane Library, PsycInfo, and LitCovid. This was followed by snowballing of the reference lists and citation search of included articles. For unpublished literature, we searched Proquest Dissertations and Theses Global, Google Scholar and various eating disorder agency websites. The search included variations of terms for COVID-19 and specific eating disorders as well as general eating concepts. Both subject headings relevant to the specific databases were employed as well as keywords. The search was further limited by including the age groups of adolescents and young adult (Table 1). The search strategy was based on previous scoping review publications [1, 6, 7, 19] and finalized through consultation with a research librarian who has a background in conducting scoping reviews.

**Table 1:** Scoping review search terms

Terms for populations	Terms for exposures	Terms for outcomes
Entry terms Adolescent Young adult	Entry terms Anorexia Binge purge Bulimia Eating disturbance Dietary restriction	Entry terms Covid Ncov Coronavirus Sars cov Hcov 2019ncov Pandemic

### Definitions

We used the following definitions for the scoping review:

- Young People: Young people were defined as early adolescence through emerging adulthood and includes individuals ages 12–29. It has been hypothesized that socioeconomic forces over the past few decades have extended the transition between childhood through adulthood into the mid- or even late 20 s and could impact the trajectories for individuals with eating disorders [54]. As such we decided to include studies where the mean age was under 30. A limitation of this approach is that some samples did contain some older individuals, however, this allowed us to include individuals in their mid-to-late 20 s (i.e., emerging adulthood).
- Restrictive Eating Disorders: Restrictive Eating Disorders were defined as anorexia nervosa or atypical anorexia nervosa and included both the restrictive and the binge-purge subtypes (when reported) [27, 55]. As Avoidant Restrictive Food Intake Disorder (ARFID) has been shown to have distinctive clinical and psychological characteristics such as being younger and exhibiting greater attention problems and clinical fears, in addition to having different treatment responses and outcomes compared to AN/AAN, we excluded it from this review [56].
- COVID-19 Pandemic: COVID-19 pandemic was defined as the pandemic that has followed the emergence of the SARS-CoV-2 virus in November 2019.

### Study selection and data extraction

Two authors independently screened the titles, abstracts, and full texts. Any discrepancies were settled through discussion. Covidence software [57] was used for screening, extracting, and analyzing the data. In adherence to the PRISMA-ScR process, a data charting form was developed and used to extract all necessary data from the final studies.

Two authors collaboratively extracted and compiled the data, which included information on author and year, study design, location and setting, sample size, patient characteristics, study outcomes, limitations, and recommendations.

### Data synthesis and analysis

A table was developed to report study details and characteristics such as year, country, study design, sample as well as study purpose. For normally distributed data such as information about the study sample, we calculated a weighted mean for normally distributed data. We reported quantitative outcomes that were statistically significant at p < 0.05. A narrative synthesis was also performed, as high levels of methodological and clinical heterogeneity indicated that a meta-analysis was inappropriate and more importantly, was not in the original scope of this review. Where possible, we synthesized quantitative data from multiple studies to identify overall effects.

## Results

**Fig. 1:**
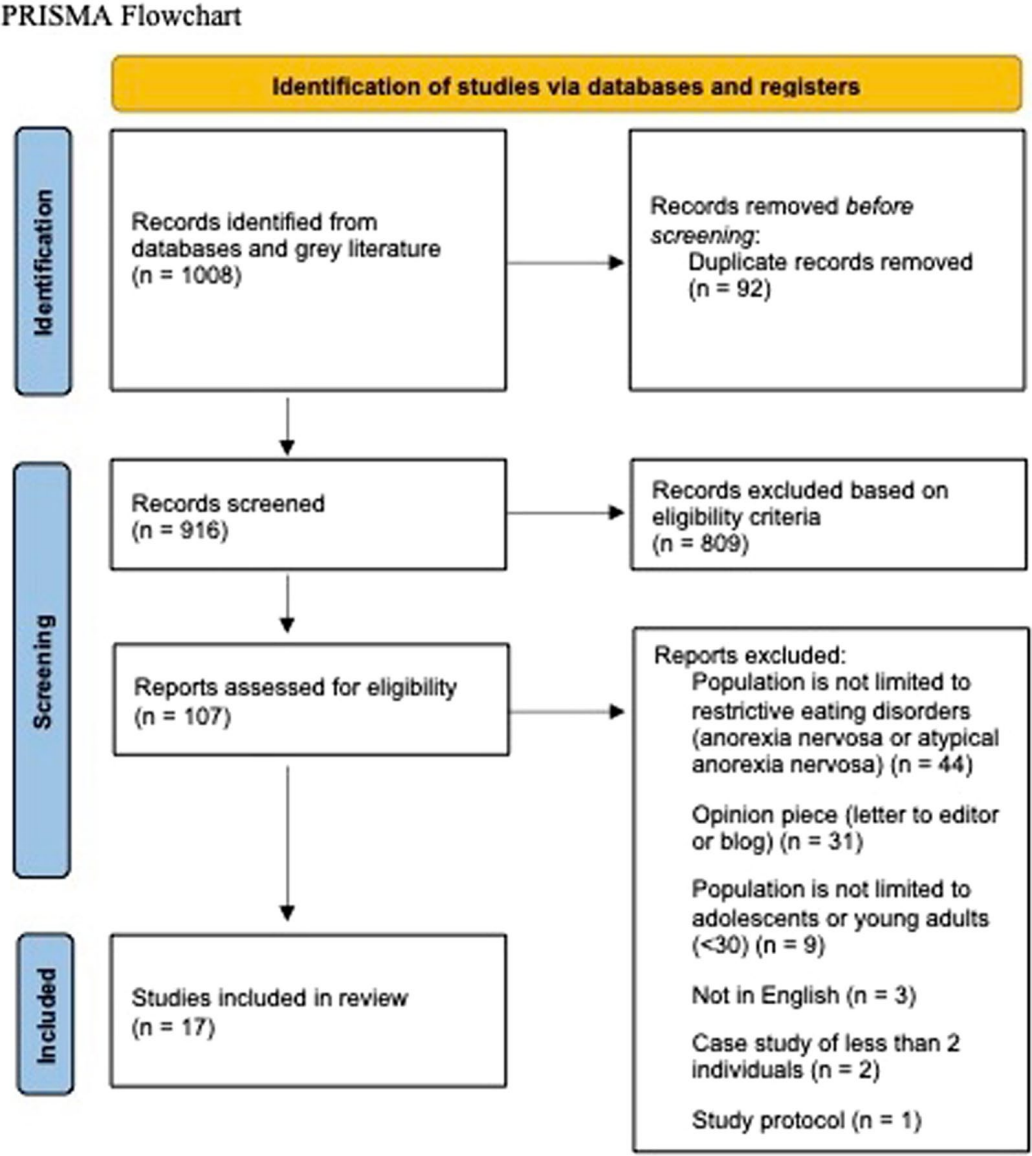
Study characteristics

From 916 unique articles, we ultimately included 17, reporting on 17 individual studies from 20 countries. These 17 articles included 4,379 individuals with individual study sample sizes ranging between 3 (case study) to 1,883. Reasons for exclusion can be found in Fig. 1. Study characteristics of these 17 papers are included in Tables 1 and 2.

**Table 2:** Study characteristics and purpose

Author	Study Characteristics			Sample	Study Purpose			
	Year	Country	Study design		Investigating Prevalence	Pandemic Impact	ED Treatment	COVID-19 Risk
Agostine et al. [71]	2015–2020	Canada	Repeated cross-sectional study comparing pandemic rates to pre-pandemic rates	1883 children and adolescents (91.0% female); median age 15.9; AN (49.2%); AAN (50.8%); race/ethnicity NR	□			
Baenas et al. 2022 [58]	2020–2021	Europe and Asia	Cross-sectional survey conducted at two time points between June 2020-January 2021	370 adolescents, young adults, and adults (80.8% female); mean age 21.9; AN (100%); AAN NR; race/ethnicity NR	□	□		
Brothwood et al. [59]	2020	UK	Qualitative, cross-sectional survey	14 adolescents (92.9% female); mean age NR range 12–18; AN (100%); AAN NR; race/ethnicity NR			□	
Fernández-Arandez et al. [60]	2020	Spain	Repeated cross-sectional study comparing pre-lockdown and post-lockdown	55 children, adolescents, young adults, and adults (89.1% female); mean age 24.2; AN (100%); AAN NR; race/ethnicity NR		□		
Goldberg et al. [72]	2015–2021	Israel	Retrospective cohort (chart-review)	275 children and adolescents (85.7% female); mean age 14.7; AN (100%); AAN (23.5%); race/ethnicity NR	□			
Hansen et al. [61]	2019–2020	New Zealand	Retrospective cohort (chart-review)	236- 86 children; 150 adults (92.3% female); mean age children- 14.6, adults—26.3; AN children (53.7%) adults (60.7%); AAN NR; race/ethnicity children- 77.5% European, adults- 93.3% European	□			
Matthews et al. [73]	2017–2020	USA	Retrospective cohort (chart-review)	163 children and adolescents (82.8% female); mean age 15.2; AN-R (7.0%); AN-BP (0.7%); AAN (8.6%); race/ethnicity- 93.3% White, 1.5% Asian, 1.2% Black	□			
Rossie et al. [62]	2019–2021	France	Pre/Post survey: pre-Covid (March 2019-March 2020), Covid (March 2020-June 20,221)	101 children and adolescents (97.0% female); mean age 14.7; AN (100%); AAN NR; race/ethnicity NR		□		
Savarese et al. [63]	2021	Italy	Cross-sectional survey	17 adolescents and young adults (100% female); mean age 18.7; AN (100%); AAN NR; race/ethnicity NR		□		
Schlegl et al. [64]	2020	Germany	Cross-sectional survey	159 adolescents and adults (100% female); mean age 22.4; AN (100%); AAN NR; race/ethnicity NR		□		
Singh et al. [65]	2015–2020	Canada	Retrospective cohort (chart-review)	425 children and adolescents (NR female); mean age 14.7; AN-R (65.6%); AAN NR; race/ethnicity NR	□			
Springall et al. [74]	2017–2020	Australia	Retrospective cohort (chart-review)	457 children and adolescents (85.8% female); mean age 14.8; AN or AAN (100%); race/ethnicity NR	□			
Tavolacci et al. [66]	2021	France	Cross-sectional survey	157 adolescents and young adults (80.5% female); mean age 20.7; restrictive ED (100%); race/ethnicity NR		□		
Taylor et al. [67]	2020–2021	UK	Cross-sectional chart review	47 adolescents, young adults, and adults (93.6% female); mean age 26.8; AN (100%); AAN NR; race/ethnicity NR				□
Ünver et al. [68]	2020	Turkey	Cross-sectional case series	3 adolescents (100% female); mean age 15; AN (100%); AAN NR; race/ethnicity NR		□		
Yaffa et al. [69]	2020	Israel	Retrospective cohort (chart-review) and cross-sectional case series	4 adolescents (100% female); mean age 17.8; AN (100%); AAN NR; race/ethnicity NR		□	□	
Zeiler et al. [70]	2020	Austria	Qualitative	13 adolescents and adults (100% female); mean age 15.9; AN-R (69.0%); AN-BP (31.0%); AAN NR; race/ethnicity NR		□		

*NR* not reported

Among the 17 studies, most study designs were observational in nature with only two qualitative studies (Table 2). In general, the studies were focused on clinical populations with two-third of the studies conducted in hospitals. Over half of studies included participants from European countries (n = 10). Among the remaining studies, three included participants from Asia, two from North America, two from Oceana, and two from countries in the Middle East. As for disease representation, 13 studies included only participants with AN [58–70] and four studies included details on individuals with AN/AAN [71–74]; only three studies specified any subtypes of AN [65, 70, 73]. Due to the infrequent reporting of whether individuals had AAN by the majority of articles, we intentionally specify if the results were from articles including AAN populations.

### Participant characteristics

Across all 17 studies, participants had a mean age of 17.6 years. Only two studies reported racial or ethnic characteristics of participants [61, 73] with different definitions of race or ethnicity, e.g., European descendent versus Caucasian/white.

### Study purpose

The majority of studies (n = 10) reported various outcomes demonstrating the impact of the pandemic on eating disorder symptomatology [58, 60, 62–64, 66, 68, 70]. Six studies compared incidence rates of AN/AAN post-onset of the pandemic to rates prior to the pandemic [58, 61, 65, 69, 71–74]. Two studies focused on eating disorder treatment outcomes [59, 69] and one study examined the risk of COVID-19 infection among patients with AN [67].

## Study findings

A wide variety of outcomes was reported by the included studies regardless of study design and participant population (Table 3). Through our analysis, we identified three prominent findings: first, there was an increase in medical hospitalizations for eating disorder-related care among young people with AN and AAN during COVID-19; second, there were changes in eating disorder-related symptomatology during the pandemic and poorer reported overall behavioral and mental health; and third, there was an increase in the use of telemedicine as a treatment modality for the treatment of AN. We also present findings on risk of COVID infection among individuals with AN.

**Table 3:** Study outcomes

Author	Outcomes			
	Hospitalization	ED-related Symptomatology	Telemedicine	Risk of COVID
Agostino et al. [71]	Monthly case increase: 60% from (24.5 to 40.6) Monthly hospitalization increase: 267% from 7.5 to 20.0)			
Baenas et al. [58]		Eating style decrease from 10.96 (SD: 8.75) to 9.98 (SD:8.71) Alcohol: decrease from 57 (15.4%) to 38 (10.3%)		
Brothwood et al. [59]			66.7% reported family sessions less helpful, 67.0% reported online individual sessions less helpful,70.0% reported meal support less helpful, and 100% reported groups less helpful (among those who previously had in-person groups) Medication reviews and dietician reviews were considered to be equally helpful online and face-to-face 71.0% felt that the program could not have done more to meet their needs Most wanted to return to face-to-face sessions for meals, family sessions, individual sessions, and group treatment sessions More than half reported being fine with continuing medication reviews and dietician reviews online Common themes were new discoveries with online treatment (more flexibility with appointments and being able to eat at home), lost in translation (e.g., technology woes/issues/lack of human aspect to treatment), and best of a bad situation	
Fernández-Aranda et al. [60]		ED symptoms decrease from 11.87 (SD: 6.79) to 9.40 (5.61), *p* = 0.015 Eating style decrease from 8.76 (SD: 9.61) to 6.11 (SD:6.94), *p* = 0.023 Emotion regulation decrease from 9.47 (SD: 4.63) to 8.33 (SD: 4.86), *p* = 0.046	AN patients found telemedicine least acceptable compared to patients with other EDs (BN, OSFED and Obesity), means (35.58, SD: 10.40) compared to BN: 28.61 (SD: 6.46), OSFED 29.50 (SD: 9.53), and obesity (28.97 (SD: 7.25)	
Goldberg et al. [72]	Yearly hospitalization increase: 208% increase (45.25 to 94) Median hospitalization duration: 35% reduction (9 days (IQR: 8.21) from 14 (6, 16.75)			
Hansen et al. [61]	Monthly hospitalization increase: 168% from 2.8 to 4.7, *p* = 0.004, for those over 18: 211% increase from 1.9 to 4			
Matthews et al. [73]	Daily admission rates not statistically different between pre-COVID lockdown and post-COVID lockdown (means went from 0.17 (SD: 0.42) to 0.24 (SD: 0.61) Daily consensus of number of inpatients increased by 154% (pre-COVID: 1.95 (SD: 1.35) to 3.02 (SD: 2.37) Rapid readmissions within 30-days of discharge 8.68 × greater likelihood post-COVID compared to pre-COVID	Readmitted patients stated causes of AN/AAN symptoms were related to gym closures/cancelling of organized sports, dieting to be healthier to avoid catching COVID-19, school closures of activity cancellations, preventing "Quarantine 15"		
Rossi et al. [62]		Sleep disturbances increased from 7.57 (SD: 5.7) to 10.49 (SD: 6.88) % with clinical insomnia (mean score > 14, increased from 7 (14%) to 17 (33.3%) Positive emotions decreased from 51.05 (SD: 12.55) to 43.38 (SD: 14.57) Happiness decreased from 23.85 (SD: 7.22) to 19.79 (SD: 7.56), Tenderness decreased from 27.2 (SD:6.60) to 23.79 (SD:7.20)		
Savarese et al. [63]		BUT-A: 100% reported weight phobia, 94.1% body image concerns, 82.4% avoidance, 88.2% compulsive self-monitoring, 100% depersonalization, and 100% global severity scale EDI-3: 82.4% risk of an ED, 5.9% are at high clinical range, 11.9% interpersonal insecurity and 100% maladjustment		
Schlegl et al. [64]		41.5% agreed or strongly agreed that the pandemic worsened their ED symptoms 70% reported that ED cognitions such as eating or shape concerns, drive for physical activity, and fear of gaining weight increased 60% reported going for a walk and at-home workouts increased More than half reported ED behaviors including restrictive eating, skipping meals, binge eating and purging were unchanged 73% maintained weight; 18.9% reported decrease (m = 4.3 kg, SD: 1.72) 51.5% reported decreased QoL More than 70% reported loneliness, inner restlessness and sadness increased Half indicated anxiety with most worries being related to COVID-19 or relapse	Phone contacts with therapist increased from 12.6% to 25.2% Videoconference therapy increased from 1.3% to 25.8% Additional online interventions increased from 3.1% to 6.9%	
Singh et al. [65]	Monthly hospitalization rate increased by 208% from 5.1 to 10.6			
Springall et al. [74]	Yearly hospitalization increase of 163% from 98.7 to 161	Reasons for AN/AAN onset included: Isolation and loneliness (32.3%), change of routine and lack of motivation (25.6%), boredom/minimal distraction from anorexic thoughts (23.3), cessation of community sport (21.1%), reduced food availability (3.0%)		
Tavolacci et al. [66]		PNNS-GS2 decreased from pre-COVID period to COVID period, mean score 4.32(SD:2.42) Percent with low or very low food security increased from 16 to 26% from pre-COVID to COVID (low- 10% to 15%, very low 6% to 11%) Frequency of never moderate physical activity (PA) decreased from 10 to 22% Frequency of never vigorous PA increased from 30 to 51% Low food security was associated with a 2.32 times increased risk of restrictive ED (1.50–3.57) Very low food security was associated with a 3.43 times increased risk of restrictive ED (2.00–5.86) Depression was associated with a 1.15 (1.12–1.18) times increased risk of restrictive ED A high PNNS-G2 score was protective and associated with a decreased risk of developing restrictive ED (0.86–0.98)		
Taylor et al. [67]				2.2% (n = 1) required hospitalization compared to 2.4–2.7% of general population
Ünver et al. [68]		Onset of symptoms occurred when COVID-19 lockdown began Social isolation was common feature. Each individual withdrew from family and peers, failed to attend online education, weighted daily, checked body in mirror, engaged in discourse around weight gain, counted calories, and spent a lot of time on social media and the internet, and prepared food at home for family members		
Yaffa et al. [69]	Outpatient care increased: from 4001 to 5926 annually (pre-COVID to COVID)		Telemedicine sessions increased from 0 (pre-pandemic) to 2192 from 1/1/20–10/31/20 37% of all sessions held were telemedicine Telemedicine was not preferred by either patients or providers Challenges such as loss of privacy during weigh checks was reported, however, "disembodied environment" created by screen, however, online treatment was adequate for 3 of 4 cases where family involvement was high	
Zeiler et al. [70]		Changes in ED and other psychopathy included: boredom and feeling of being observed (especially during weight checks) triggering eating disorder AN-symptoms, restrictive behaviors such as calorie counting, obsessive exercising and binge-purging behaviors AN-related cognitions and behaviors were also increased by social comparisons and exchanges with peers/friends COVID-19-related fears and compulsions such as fear about health of relatives and concern over ability to purchase certain food items were expressed Some reported general improvements in symptoms indicating a normal treatment course	Challenges and opportunities of teletherapy/telemedicine although therapy was perceived as "rather satisfactory" however face-to-face was still preferred Challenges of having therapy at home meant that participants felt problems were always around them and lack of privacy in appointments because of concern someone may overhear conversation	

*BN* Bulimia Nervosa, *BUT-A* Body Uneasiness Test Part A, *EDI-3* Eating Disorder Risk scale, *IQR* interquartile range, *ISI* insomnia severity index, *OSFED* other specified feeding or eating disorder, *PNNS-GS2* Programme National Nutrition Santé, *SD* standard deviation

### Changes in hospitalization rates

One of the most salient findings was the increase in medical hospitalizations for patients with AN/AAN after the onset of the COVID-19 pandemic compared to pre-onset of the pandemic. Individuals were more frequently hospitalized for medical instability even though the average duration of disease was shorter [65, 71, 73]. While there were significant increases in hospitalization rates, both monthly (between 1.68–2.68 times greater) [61, 65, 71] and yearly (between 1.63–2.08 times greater) [72, 74], the median length of hospitalization stay was found to decrease by 35% [72]. One study found the number of inpatients increased by 1.5 times greater, however, the increase was not statistically different than admission rates pre-COVID-19 [73]. Furthermore, individuals discharged after the onset of the COVID-19 pandemic were found to be 8.7 more likely to be rapidly readmitted, for example, within 30-days, compared to individuals who had been discharged before the onset of the COVID-19 pandemic [73]. As for outpatient care, monthly caseloads were found to increase by 1.48–1.66 times greater compared to pre-COVID-19 [69, 71].

However, two studies did not find increases in medical usage among individuals with AN/AAN. Springall et al. [74] found fewer patients with AN presenting with severe malnutrition post-onset during post-pandemic (e.g., after 2020) compared to pre-pandemic (2017–2019) and Goldberg et al. [72] did not find differences in the number of patients hospitalized with AN requiring nasogastric tube feeding; a measure often used to indicate disease severity.

### Changes in eating disorder-related symptomology

Over half of included studies reported changes in eating disorder-related symptomatology among young people with AN/AAN [58, 60, 62–64, 66, 68, 70, 73, 74]. In general, the COVID-19 pandemic was found to negatively impact a variety of eating disorder symptomatology including increased anorexia-related cognitions and behaviors [63, 64, 68, 70, 74]. An increase in drive for physical activity was reported by one study with over 60% of respondents reporting increased rates of going for a walk and at-home workouts in another study [64]. Additionally, more than half of respondents in that study indicated increases in eating disorder cognitions such as drive for thinness, fear of gaining weight, body dissatisfaction, eating concerns, shape concerns, weight concern [64]. Suggested reasons behind changes in increased anorexia-related behaviors included boredom or minimal distraction from pathological thoughts, increased social isolation, increased social media and online use (e.g., reading blogs or watching YouTube), gym and school closures, changes in routines due to lockdowns and quarantines, and worries over gaining “Quarantine 15” (i.e., 15 pounds that were associated with the COVID-19 pandemic) [64, 68, 70, 73, 74]. Furthermore, over half of respondents reported their eating disorder giving them a sense of control or safety [64].

The COVID-19 pandemic was also found to impact food obsessions and rituals, a key aspect of AN, which can include diet and food intake. Among individuals with AN, decreased food intake (i.e., potentially increased food restriction), was found during COVID-19 compared to pre-COVID-19 [58, 60, 66, 74]. Decreased availability of desired foods, which can especially be important during recovery, was also reported during Covid-19 [58, 60, 66, 74]. Similarly, decreased food security was also found during the pandemic [58, 60, 66, 74]. Furthermore, reporting food security was associated with a decreased risk of developing a restrictive eating disorder [66].

Two studies found mixed results on how the COVID-19 pandemic impacted eating disorder symptomatology with Zeiler et al. [70] finding general improvements in symptoms during Covid-19, such as more regular eating due to always being home during lockdowns. Schlegl et al. [64] also reported themes such as how COVID-19 was a “wake-up call/ [reminded them of their] will to live”, with one individual reporting using a two-week quarantine period as a way to “stop my excessive exercise behavior”, and that pandemic helped them with “accepting uncertainty in life”.

The COVID-19 pandemic was also associated with poorer overall behavioral and mental health among young people with AN/AAN. Increased sleep disturbances and a decreases in emotional regulation after the onset of the COVID-19 pandemic were reported by multiple studies [60, 62]. Increased depression, loneliness, and compulsions around concerns over the health of relatives were also reported among this population [64, 66, 70, 74]. Furthermore, the COVID-19 pandemic was found to impact anxious thoughts, especially increased worries around food security as well as increased AN-specific and COVID-19 pandemic-specific anxieties [64, 66, 70, 74].

### Use of telemedicine as treatment during Covid-19

Our third finding was that telemedicine, often defined as videocalls through an online platform, was largely accepted during the COVID-19 pandemic, even though some studies found patients and providers reported negative experiences. While some providers and programs had previously used telemedicine for the treatment of eating disorders, in general telemedicine was a new modality [59, 64, 69]. Patients reported that the increased flexibility with appointments and being able to eat meals at home–a key component of eating disorder treatment–were benefits of telehealth [59]. Ultimately, individuals with AN/AAN felt that telemedicine was the best option during COVID-19 lockdowns when compared to no treatment; however, some individuals, especially those who had received past treatment, still expressed the preference for in-person treatment when available [59, 69, 70].

Regardless of history of past usage of telemedicine, challenges were reported by both patients and providers with some patients expressing a desire to return to in-person treatment [59, 60, 64, 69, 70]. When comparing individuals with AN to individuals with other eating disorders (e.g., individuals with bulimia nervosa, other specified feeding or eating disorder (OSFED) or other weight-related outcomes (i.e., obesity)), individuals with AN found telemedicine to be less acceptable compared to other treatment modalities, such as in-person [60]. Furthermore, while group therapy can be an important component of AN/AAN treatment [75], numerous challenges were reported to group therapy in an online format. Online group therapy was not viewed to be the same as in-person group therapy as the online environment lacked the “human aspect” and did not facilitate building connections between members, such as informal conversations between patients as well as between patients and providers that happen during the setting up for group therapy [59]. Additional challenges mentioned by patients included privacy concerns during therapy appointments (i.e., the concern that family-members could hear what was being discussed) and lack of privacy during weight and other vital checks, as well as general technology issues such as connectivity challenges [69, 70].

### COVID-19 infection risk

Taylor et al. [67] was the only study to report on the risk of infection with the SARS-Cov-2 virus in patients with AN/AAN. This study found that having a restrictive eating disorder does not put an individual at increased risk of developing an infection from COVID-19. Most participants presented with typical COVID-19 symptoms (e.g., fever, cough, or loss of smell or taste) with 93.6% experiencing mild disease. Among those who experienced more severe infection (n = 3), two developed pneumonia, and one required hospitalization for treatment due to respiratory-related symptoms.

## Discussion

We conducted a scoping review to identify the current knowledge and gaps in the field around how the COVID-19 pandemic impacted young people with restrictive eating disorders around the world. Our scoping review examined 916 studies from 2019 to 2022, with 17 studies included in our final review and analysis. Our overall findings indicate that COVID-19 significantly impacted young people with restrictive eating disorders in various ways.

Our first finding is that, in general, medical hospitalizations for young people with AN/AAN increased during COVID-19, mirroring findings published by other studies [21]. While few peer-reviewed studies were published in the first two years of COVID-19, many editorials and letters to the editor were published addressing the rise in the number of hospitalizations and changes in eating disorder-related symptomatology [18, 76–85]. During the first year of the COVID-19 pandemic in Australia, a 104% increase in child AN-related medical hospitalizations compared to the prior three pre-pandemic years was observed [18]. In Ireland, a 66% increase in admissions in children with eating disorders was found in 2020 compared to 2019 with children presenting with lower BMIs and generally more medically compromised and less stable [80]. The number of individuals requiring in-patient treatment for AN more than doubled in England in 2020 [81]. Increases were also found in other non-Western countries including Pakistan and Singapore; however, the authors did not differentiate between types of eating disorders [86, 87]. Though increased recognition may be one contributor, as many families spent increased time together during lockdowns and quarantines, it is unlikely that this fully explains the marked increase in hospitalizations.

Our second finding is the changes in eating disorder-related symptomatology with many individuals with AN/AAN reporting increased symptoms after the onset of the COVID-19 pandemic. A qualitative study with adults with AN during COVID-19 found similar findings with individuals reporting difficulties accessing “safe” food, increase in anxiety and stress, as well as exacerbation of disordered thoughts and behaviors happening during lockdown [88]. While this study was with adults, it mirrors the findings of the current study, in particular, how lockdowns impacted the mental health of individuals with AN. The role that lockdowns played in the development and/or exacerbation of restrictive eating disorders should not be underestimated [89] and we will not know the full impact of such lockdowns for years to come.

Our third and final finding is the increase in the use of telemedicine as a treatment modality during the COVID-19 pandemic but not without challenges to both patients and providers. Telemedicine, also called telehealth, existed prior to the onset of the pandemic, but with the implementation of quarantines and lockdowns, providers rapidly shifted to giving care through telemedicine as a way to continue to see non-urgent patients [90].

Despite the previously discussed limitations, telemedicine did result in positive outcomes among those with AN. Raykos et al. found that therapy delivered through tele-health resulted in an improvement in both symptoms and overall eating disorder disease among patients with eating disorders in Australia [91]. Another study in the United States found improvements in overall eating disorder symptoms, depression, and perfectionism regardless of whether programming was delivered via telehealth or in-person [92]. Furthermore, BMIs significantly increased regardless of treatment modality [92]. While these do not contradict our results, it is important to consider the challenges mentioned by patients and providers alike. Similar to the challenges identified in this review, the ability to develop rapport and to successfully monitor and conduct assessments through telehealth during quarantine was limited compared to prior in-person treatment [88, 91, 92]. It has been argued that these challenges could potentially undermine both the therapeutic relationship as well as the overall efficacy of treatment [93]. While young people are often thought of as being tech-savvy, our results highlight the challenges with using telehealth and how impersonal it can be. There has also been sharing of lessons learned as Waller et al. among others, published recommendations to assist in decreasing barriers when delivering teleservices for patients with eating disorders [91, 94–97]. Future research examining screening and prevention using telemedicine could benefit treatment of individuals suffering from this disorder.

This review was able to address how the COVID-19 pandemic impacted young people with AN/AAN by finding increased rates of AN/AAN among young people and that, in general, the pandemic has worsened eating disorder-specific disease symptoms. The COVID-19 pandemic created additional stress to a highly burdened and thinly stretched healthcare systems thus potentially creating additional challenges to accessing care for young people with AN/AAN. As providers pivoted to provide treatment through telemedicine, it will be interesting to see if and how widely this modality is used. As emergency measures in the United States that allowed for providers to treat patients across state lines have begun to expire, some of the benefits of telemedicine as a treatment modality may be unavailable.

How the COVID-19 pandemic specifically impacted young people with AN/AAN may not be known for years to come. Increased screen time [98, 99] and school closures [100] have both been associated with poorer mental health among young people. Adolescents and children have been further hypothesized to be at increased risk of developing anxiety and depression, both well-documented comorbid conditions among individuals with AN and AAN and it is unknown how long these conditions may last [10]. Decreased time spent with peers and in social settings may impact development of young people over time [100]. This is especially concerning as the rates of screen usage have remained elevated and screens may be used as a coping mechanism or as a way to withdraw from stressors [99]. Lastly, while it is known that in general, young people, lack the same degree of resilience and coping compared to adults, it is unclear how lower resilience may impact the disease trajectories of young people with AN/AAN going forward [6].

This review has several important limitations. First, the number of relevant published studies was limited and a large proportion of them were poorly designed resulting in low generalizability. Second, the majority of the studies were observational in nature and focused on clinical samples [101]. Third, the majority of countries included in this scoping review were Western Countries with well-established healthcare systems such as those in Europe, North America and Oceana, and there were no studies from Africa and South America. Additionally, only one study included three Asian countries, and two studies included one Middle Eastern country each (Turkey and Israel). While most of the countries included in this review are represented in the eating disorder field, it is unclear how the pandemic may have impacted other less studied nations. Fourth, the majority of studies were affiliated with hospitals or treatment centers, thus potentially underreporting the true cases/counts by missing individuals in the community. Fifth, demographics were missing from most studies; 88% of studies did not include information on the racial or ethnic background of participants, so it is therefore impossible to identify whether certain racial/ethnic groups are missing, as it is possible that some individuals were unable to seek treatment. Moreover, barriers to accessing treatment for individuals of lower socioeconomic status are known and this study is unable to examine how COVID-19 may have specifically impacted this population. It is also not possible to determine whether additional marginalized populations, such as LGTBQ + , certain racial or ethnic groups including indigenous people, as well as immigrants or refugees are included. Sixth, most studies were conducted in urban environments, so generalizability to rural populations cannot be made.

Another important limitation is that there were many differences in how countries handled the COVID-19 pandemic. The enforcement, definition, and length of lockdowns varied from nation to nation. Different countries responded to the pandemic in different ways with diverse lockdowns and quarantines. Over-burdened health care systems and exhausted medical providers were common as the treatment of chronic conditions were delayed due to COVID-19 concerns. It is unclear how increased recognition from the family and thus earlier identification of eating disorders may be driving rates of eating disorders [101]. Furthermore, the availability of inpatient beds or dedicated ED treatment services may differ between different countries thus impacting the rates of hospitalizations. Even with such limitations, this scoping review addresses a key gap in the current literature.

## Conclusions

We conducted a comprehensive scoping review of the impact of the COVID-19 pandemic on young people with AN/AAN. We found that limited studies with heterogenous study designs and unclear populations exist. Most studies to date have been associated with hospital or clinical setting in Western Nations. We found increased rates of AN and AAN among young people and that, in general, the pandemic has worsened eating disorder-specific disease symptoms thus impacting an already burdened and thinly stretched healthcare system. We also found that while telehealth may not be preferred by all, it may work, and future studies focused on how to improve telehealth are warranted.

While we found that there has been some study in this arena, significant gaps still exist. We want to acknowledge that the number of publications included in this review, 17 in the past few years, reflect the perceived importance of examining the COVID-19 pandemic effects on young people with AN/AAN by clinicians and researchers alike. Further research is needed to examine the long-term implications the COVID-19 pandemic has had on this population and to determine whether the disease trajectory remains similar for young persons diagnosed or treated during the early years of the pandemic. Further research is also needed to examine how the series of lockdowns and subsequent re-openings have impacted this population.

## Data Availability

All data is published and in the public domain. Data sharing is not applicable to this article as no datasets were generated or analyzed during the current study.

## Abbreviations

AN: Anorexia Nervosa
AAN: Atypical Anorexia Nervosa
BN: Bulimia Nervosa
BUT-A: Body Uneasiness Test Part A
EDI-3: Eating Disorder Risk scale
IQR: Interquartile range
ISI: Insomnia severity index
OSFED: Other specified feeding or eating disorder
PNNS-GS2: Programme National Nutrition Santé
COVID-19: SARS-COVID-19 global pandemic
SD: Standard deviation
